# Prenatal depression among pregnant women attending public health facilities in Babile district, Eastern Ethiopia: a cross-sectional study

**DOI:** 10.1186/s12888-024-05732-0

**Published:** 2024-05-07

**Authors:** Sherif Jibrael Ahmed, Melkamu Merid, Dumessa Edessa, Ahmedin Aliyi Usso, Hassen Abdi Adem, Mandaras Tariku, Abdulbasit Seid, Addisu Alemu, Addis Eyeberu, Mohammed Yuya

**Affiliations:** 1 East Hararghe Zone Health Office, Oromia Regional Health Bureau, Federal Ministry of Health, Harar, Ethiopia; 2 School of Public Health, College of Health and Medical Sciences, Haramaya University, Harar, Ethiopia; 3 School of Pharmacy, College of Health and Medical Sciences, Haramaya University, Harar, Ethiopia; 4 School of Nursing and Midwifery, College of Health and Medical Sciences, Haramaya University, Harar, Ethiopia; 5 Department of Psychiatry, College of Health and Medical Sciences, Haramaya University, Harar, Ethiopia; 6 School of Public Health and Preventive Medicine, Monash University, Melbourne VIC 3004 , Australia

**Keywords:** Perinatal, Prenatal depression, Antenatal, Pregnancy, Health facilities, Ethiopia

## Abstract

**Background:**

Depression during pregnancy is a significant health concern that can lead to a variety of short and long-term complications for mothers. Unfortunately, there is a lack of information available on the prevalence and predictors of prenatal depression in rural eastern Ethiopia. This study assessed prenatal depression and associated factors among pregnant women attending public health facilities in the Babile district, Eastern Ethiopia.

**Method:**

An institution-based cross-sectional study was conducted among 329 pregnant women attending Babile District Public Health Facilities from November 1 to December 30, 2021. Bivariable and multivariable logistic regression were used to identify factors associated with prenatal depression. The adjusted odds ratio (AOR) with a 95% confidence interval was used to report the association, and the significance was declared at a p-value < 0.05.

**Results:**

The prevalence of prenatal depression was 33.1% (95% CI = 28.0%, 38.2%). A lower income (AOR = 3.85, 95% CI = 2.08, 7.13), contraceptive use (AOR = 0.53, 95% CI = 0.28, 0.98), unintended pregnancy (AOR = 2.24, 95% CI = 1.27, 3.98), history of depression (AOR = 5.09, 95% CI = 2.77, 9.35), poor social support (AOR = 5.08, 95% CI = 2.15, 11.99), and dissatisfied marriage (AOR = 2.37, 95% CI = 1.30, 4.33) were the factors associated with increased prenatal depression among pregnant women.

**Conclusions:**

One in every three pregnant women in rural eastern Ethiopia had prenatal depression. Monthly income, contraceptive use, pregnancy intention, history of depression, social support, and marriage satisfaction status were the determinants of prenatal depression. Preventing unintended pregnancies by encouraging women to utilize modern contraceptive methods is essential for mitigating and controlling the risks and burdens of prenatal depression and its negative consequences.

## Introduction

Depression is a mood disorder characterized by persistent feelings of low self-worth, loss of interest, feelings of regret, restlessness, loss of appetite, fatigue, and poor concentration [1–3]. Prenatal depression is a depression that occurs during the time from conception to delivery and is a precursor of postpartum depression if it remains untreated or mismanaged [4]. It is the most prevalent psychiatric disorder experienced during pregnancy, and almost 25% of women experience depression at some point in their life, most commonly during childbearing age [5, 6].

Prenatal depression is a global burden of disease and affects approximately 10% of pregnant women each year [5]. The burden of prenatal depression is 25.5% in lower- and middle-income countries (LMICs) [7]. The burden is also higher than that in sub-Saharan Africa (SSA), which ranges from 10 to 47% [8–10], and the prevalence of prenatal depression ranges from 12 to 35% in Ethiopia [10–14].

Prenatal depressive disorders can lead to various short and long-term fetal and maternal negative consequences, including low birth weight, intrauterine growth restriction, preterm birth, and stillbirth, and can also contribute to maternal and neonatal disability, morbidity, and mortality [15–17]. In addition, it negatively influences social adjustment and marital relationships [18, 19] and affects maternal-infant interactions through its influence on the occurrence of postnatal depression [20, 21].

Prenatal depression has a significant potential effect on the economy, and it is more susceptible to adverse effects through its impact on healthcare costs and work absenteeism, which accounts for one-third of the work days missed and one-fifth of all primary healthcare appointments missed [22]. Although reports on prevalence rates lack consistency, substantially higher rates were reported in developing countries [5].

There are various factors affecting prenatal depression, but little is known about these factors in developing countries, including Ethiopia. The studies indicated that factors such as low economic status, previous history of psychiatric illness, abortion history, and previous stillbirth history were determinants of prenatal depression [23–26]. The government of Ethiopia launched and enforced a mental health strategy that aimed at providing mental health services in all health systems of the country [27]. However, there is a gap in providing mental health services in routine maternal health services. Even though investigating prenatal depression and its risk factors is essential for designing and monitoring the implementation of preventive and rehabilitative interventions designed to reduce the impacts of depression, the evidence regarding prenatal depression is inconsistent in Ethiopia.

Hence, addressing the gaps in burdens and risk factors for prenatal depression among pregnant women is a top priority in reducing and preventing prenatal depression and its negative short- and long-term consequences. Previous studies have shown that the burden of prenatal depression is inconsistent in Ethiopia [11, 23], and there is limited information available concerning this topic in rural eastern Ethiopia. In addition, previous studies conducted in the country were performed in a one-site region of Ethiopia, and almost all the studies failed to evaluate the association between prenatal depression and the use of contraceptive methods. Overall, there is limited information on the prevalence and factors associated with prenatal depression among pregnant women in rural Eastern Ethiopia. Thus, this study aimed to assess the level of prenatal depression and associated factors among pregnant women attending public health facilities in the Babile district, eastern Ethiopia.

## Materials and methods

### Study design and setting

An institution-based cross-sectional study was conducted in the Babile district, eastern Ethiopia, from November 1 to December 30, 2021. The Babile district is one of 24 districts in the East Hararghe Zone, located 560 km, east of Addis Ababa (the capital city of Ethiopia). Administratively, the district had one urban and 23 rural kebeles, and based on the 2007 Central Statistical Agency population census, the district has a projected total population of 103,575, with 22,921 reproductive-age women and 3594 pregnant women. According to the Babile District Health Office annual report of 2020, there is one general hospital, four health centers, and 24 health posts, with two private drug shops providing routine healthcare services for the general public in the district. The annual antenatal care (ANC) attendance in the facilities of the district was 6780 in the Babile Health Center, 1020 in the Abdibuch Health Center, 1320 in the Erer Health Center, and 1500 in the Bisidimo General Hospital.

### Population and sampling

All pregnant women attending public health facilities in the Babile district in Eastern Ethiopia were the source population. The study population included pregnant women attending selected public health facilities in the Babile district in Eastern Ethiopia during the study period. Married pregnant women aged 18 years and older who were permanent residents of the district and who attended public health facilities for antenatal care service were included in the study. Women who were unable to understand the local language (Afan Oromo), who were critically sick pregnant women, or who were urgently or immediately referred to a higher-level facility upon arrival at the interview were excluded from the study.

The sample size (*n* = 330) was calculated by Epi Info version 7.1 using the single population proportion formula. Considering the following assumptions, the average monthly ANC attendance in the district was 565, the confidence interval was 95%, the margin of error was 5%, the prevalence of prenatal depression was 26.6% in southern Ethiopia [28], and the nonresponse rate was 10%.

We selected purposefully all public health facilities (four health centers and one hospital) in the district. The projected sample size was proportionally allocated to each facility based on the average ANC attendance flows over the previous three months. The sample sizes were proportionally allocated to the Babile Health Center (*n* = 90), Abdibuch Health Center (*n* = 50), Erer Health Center (*n* = 64), Awsherif Health Center (*n* = 53), and Bisidimo General Hospital (*n* = 73). Then, a systematic random sampling technique was used to select the study participants, and every three eligible women who visited ANC facilities were selected from among the health facilities. After providing written informed consent, a face-to-face interview was conducted in a separate and quiet area.

### Data collection procedures and tools

The data were collected through face-to-face interviews using a structured questionnaire adapted from validated scales and published literature [23, 25, 29–35]. The questionnaire included information on sociodemographic characteristics, obstetric-related characteristics, behavioral characteristics, mental health and psychosocial characteristics, and prenatal depression. Before data collection began, the questionnaire was pretested on 5% of the sample (17 pregnant women) in a separate health facility in the neighboring district to ensure its validity. The questionnaire was first prepared in English, subsequently translated to the local language (Afan Oromo), and returned to English by two experts who were in good command of both languages. Five trained diploma nurses collected the data through face-to-face interviews in a quiet private room. The overall data collection process was supervised by trained BSc nurses at each facility.

### Measurements

#### Prenatal depression

The Edinburgh Postnatal Depression Scale (EPDS) was used to detect prenatal depression [32]. The EPDS is a validated scale with 10 Likert scale items, each rated from 0 (mild depression) to 3 (high depression), for detecting depression in antepartum and postpartum samples across the world; this scale includes information from Ethiopia (postpartum depression assessment in Addis Ababa with a sensitivity of 80.6% and a specificity of 77%) [29]. Similar to other studies conducted abroad and in Ethiopia, the cutoff point of the EPDS was 13 and above to identify pregnant women with depressive symptoms. The internal consistency of the index score was confirmed with a Cronbach’s α of 0.92. Pregnant women who scored 13 or above were categorized as depressed women, while those who scored less than 13 were categorized as not depressed women [25].

#### Level of social support

Social Support was measured using the Oslo Social Support Scale-3 item (OSSS-3). The OSSS-3 contains three items: the number of close intimates, the perceived level of concern from others, and the perceived ease of getting help from neighbors. The composite index score was computed from three items ranging from 3 to 14, and OSSS-3 scores were categorized into three categories of social support. The internal consistency of the index score was confirmed with a Cronbach’s α of 0.76. The scores were subsequently assigned as 3–8 for ‘poor social support’, 9–11 for ‘moderate social support’, and 12–14 for ‘strong social support’ [30].

#### Marital satisfaction

This was measured using the Kansas Marital Satisfaction Scale (KMSS), which contains three Likert scale items. The marriage satisfaction scale included three items, each of which had a 7-point scale ranging from 1 (extremely dissatisfied) to 7 (extremely satisfied*);* the composite index score was computed from three items ranging from 3 to 21. The internal consistency of the index score was confirmed with a Cronbach’s α of 0.83. A cutoff point of 17 and above was used to indicate that women were satisfied with their current marital relations, while a cutoff point less than 17 was used to indicate that women were dissatisfied with their current marital relations [31, 33].

**Substance use** was assessed using a self-report of exposure to at least one of the three substances (alcohol, khat, or tobacco) during the current pregnancy. A pregnant woman was considered to use a substance when she used at least one type of substance during her current pregnancy, regardless of the dose and frequency [36].

### Data quality control

The data quality was maintained using standard questionnaires adapted from validated scales and relevant published literature. The questionnaires were first written in English and subsequently translated into the local language (Afan Oromo) and returned to English by two experts with good mastery of both languages. The data collectors and supervisors trained for one day on the objective of the study and the data collection technique. Before starting the statistical analysis, several composite index scores were computed and used accordingly, which improved the validity of the measurements and respective computed indices and estimates used in the study.

### Data processing and analysis

After checking for completeness and consistency, the data were entered into EpiData version 3.1 and analyzed using SPSS version 24. Descriptive statistics such as frequency, a measure of central tendency, and dispersion were used to characterize pregnant women accordingly. Before analysis, the internal consistency of the items was checked for each composite index score using reliability analysis (Cronbach’s α). We observed high internal consistency across all the composite indices of the EPDS use items (Cronbach’s α = 0.92), the KMSS use items (Cronbach’s α = 0.83), and the OSSS use items (Cronbach’s α = 0.76). Bivariable and multivariable logistic regression analyses were also conducted to identify factors associated with prenatal depression among pregnant women. Variables with a p-value < 0.25 in the bivariable analysis were selected as candidate predictors for multivariable logistic regression analysis. A multivariable logistic regression analysis was conducted to identify the significant risk factors for prenatal depression using the backward stepwise likelihood ratio method for model building. The Hosmer and Lemeshow goodness-of-fit test was employed to determine the overall adequacy of the model, with a p-value of 0.65 indicating an adequate fit. The adjusted odds ratio (AOR) with its 95% confidence interval (CI) was used to report the strength of an association, and a p-value < 0.05 indicated statistical significance.

## Results

### Participants sociodemographic characteristics

A total of 330 eligible pregnant women were invited to participate in the study, and 329 (99.7%) participated in the study. The mean age of the participants was 29.2 (± 5.6) years, and the majority (65.5%) of them were in 25–34 years age group, followed by 27.4% in the 18–24 years age group and 26.1% in the ≥ 35 years age group. More than half (55.0%) of the participants were from rural residences. Approximately half (49.5%) of the participants had an education level of primary school, followed by being unable to read and write (36.8%), secondary school (9.7%), and college and above (4.0%). Among occupational status, the majority (63.5%) of the participants were housewives, followed by merchants (18.2%) and government employees (13.1%). Regarding average monthly income, a majority (54.1%) of the participants had a monthly income less than or equal to 2000 Ethiopian Birr (ETB), and their median average monthly income was 2000 ETB (quartile 1 = 1200 ETB, quartile 3 = 3200 ETB) (Table 1).

**Table 1:** Sociodemographic characteristics of pregnant women in the Babile district, eastern Ethiopia (*n* = 329)

Variables	Categories	Frequency	Percent
Resident area	Rural	181	55.0
	Urban	148	45.0
Age (complete year)	18–24	90	27.4
	25–34	150	65.5
	≥ 35	86	26.1
Ethnicity	Oromo	271	82.4
	Somale	38	11.6
	Amhara	14	4.3
	Gurage	6	1.8
Religion	Muslim	281	85.4
	Orthodocs	38	11.6
	Protestant	10	3.0
Educational status	Unable read and write	121	36.8
	Primary school	163	49.5
	Secondary school	32	9.7
	College and above	13	4.0
Husband educational status	Unable read and write	103	31.9
	Primary school	145	44.9
	Secondary school	39	12.1
	College and above	36	11.1
Occupational status	House wife	209	63.5
	Merchant	60	18.2
	Govn’t employee	43	13.1
	Private employee	11	3.3
	Others^a^	6	1.8
Husband occupational status	Farmer	194	60.1
	Merchant	65	20.1
	Govn’t employee	48	14.9
	Private employee	10	3.1
	Others^a^	6	1.9
Average monthly income (in ETB)	≤ 2000	178	54.1
	> 2000	151	45.9

Note: a, daily laborers, students; ETB, Ethiopian Birr

### Obstetrics and psychosocial-related characteristics

The means (± SD) gravidity and parity were 4.8 (± 2.4) and 3.9 (± 2.1), respectively. Almost one in every five (21.1%) women had at least one abortion history, and one in every four (25.3%) participants had at least one stillbirth history. The majority (59.6%) of the women had never used modern contraceptive methods before their current pregnancies, and approximately 38.9% of the current pregnancies were unintended pregnancies. The majority (58.7%) of women were in the second trimester, followed by the third trimester (33.1%) and the first trimester (8.2%). Approximately 15.5% of pregnant women used at least one type of substance during their current pregnancy. About 31.6% of the pregnant women were violated by their partners, and approximately 32.8% of the pregnant women had a history of depression. Regarding social support, 41.6%, 41.0%, and 17.3% of the pregnant women had poor, moderate, and strong social support, respectively. Almost seven out of ten pregnant women (69.3%) were satisfied with their marriage relationship (Table 2).

**Table 2:** Obstetrics and psychosocial characteristics of pregnant women in the Babile district, eastern Ethiopia (*n* = 329)

Variables	Categories	Frequency	Percent
Gravidity	1	44	13.4
	2–4	148	45.0
	≥ 5	137	41.6
Parity	0	44	13.4
	1–4	175	53.2
	≥ 5	110	33.4
Abortion history	No	225	78.9
	Yes	60	21.1
Stillbirth history	No	213	74.7
	Yes	72	25.3
Birth complication history	No	189	66.3
	Yes	95	33.7
Modern contraceptive use	Yes	133	40.4
	No	196	59.6
Pregnancy intention	Unintended	128	38.9
	Intended	201	61.1
Trimester	First	27	8.2
	Second	193	58.7
	Third	109	33.1
Substance use	Yes	51	15.5
	No	278	84.5
Parents violence	Yes	104	31.6
	No	225	68.4
Depression history	No	108	32.8
	Yes	221	67.2
Level of social support	Poor	137	41.6
	Moderate	135	41.0
	Strong	57	17.3
Marriage relation status	Dissatisfied	101	30.7
	Satisfied	228	69.3

### Prevalence of prenatal depression

In this study, the internal consistency of the prenatal depression (EPDS) tool was acceptable, with Cronbach’s α = 0.92. The overall prevalence of prenatal depression (EPDS score ≥ 13) among pregnant women was 33.1% (95% CI = 28.0%, 38.2%) in the Babile district, eastern Ethiopia.

### Factors associated with prenatal depression

The bivariable logistic regression analysis showed that average monthly income, gravidity, current pregnancy intentions, trimester, substance use, parental violence, history of depression, level of social support, and marriage satisfaction status were factors significantly associated with prenatal depression. However, in the multivariable logistic regression analysis, average monthly income, use of contraceptives, pregnancy intention, history of depression, level of social support, and marriage satisfaction status were the main predictors of prenatal depression among pregnant women.

Women who had an average monthly income less than or equal to 2000 ETB were almost four times more likely to be depressed during pregnancy (AOR = 3.85, 95% CI: 2.08, 7.13) than those who had an average monthly income greater than 2000 ETB. The women who used contraceptive methods before their current pregnancy were almost 50% less likely to develop prenatal depression during pregnancy (AOR = 0.53; 95% CI = 0.28, 0.98). The odds of prenatal depression were approximately two times greater among women with unintended pregnancies than among those with planned pregnancies (AOR = 2.24, 95% CI = 1.27, 3.98). Women who had a history of previous depression were five times more likely (AOR = 5.09, 95% CI = 2.77, 9.35) to develop prenatal depression than those who had no previous history of depression. Pregnant women who had poor social support were five times more likely to develop prenatal depression than those who had strong social support (AOR = 5.08, 95% CI = 2.15, 11.99). The odds of prenatal depression were approximately two times greater (AOR = 2.37, 95% CI: 1.30, 4.33) among women dissatisfied with their marriage than among women who were satisfied with their marriage (Table 3).

**Table 3:** Factors associated with prenatal depression among pregnant women in Babile District, eastern Ethiopia (*n* = 329)

Variables	Categories	Prenatal depression		COR (95% CI)	AOR (95%CI)
		Yes n(%)	No n(%)		
Residence	Rural	64 (35.4)	117 (64.6)	1.25(0.79, 1.99)	1.20(0.61, 2.37)
	Urban	45 (30.4)	103 (69.6)	1	1
Income (in ETB)	≤ 2000	80 (44.0)	98 (55.1)	3.43(2.08, 5.67) ***	3.85(2.08, 7.13) ***
	> 2000	29 (19.2)	122 (80.8)	1	1
Gravidity	1	12 (27.3)	32 (72.7)	1.05(0.49, 2.26)	1.67(0.41, 3.31)
	2–34	61 (41.2)	87 (58.8)	1.97(1.19, 3.25)	1.69(0.84, 3.40)
	≥ 5	36 (26.3)	10 (73.7)	1	1
Ever used contraceptive	Yes	45 (33.8)	88 (66.2)	1.05(0.66, 1.68)	0.53(0.28, 0.98) *
	No	64 (32.7)	132 (67.3)	1	1
Current pregnancy intention	Unintended	58 (45.3)	70 (54.7)	2.44(1.52 3.90) ***	2.24(1.27, 3.98) **
	Intended	52 (25.4)	150 (74.6)	1	1
Trimester	First	10 (37.0)	17 (63.0)	1.70(0.70, 4.15)	2.14(0.69, 6.63)
	Second	71 (36.8)	122 (63.2)	1.68(1.00,2.83) *	1.60(0.83, 3.05)
	Third	28 (25.7)	81 (74.3)	1	1
Substance use	Yes	25 (49.0)	26 (51.0)	2.22(1.21, 4.07) *	1.58(0.74, 3.35)
	No	84 (30.2)	194 (80.8)	1	1
Parent’s violence	Yes	48 (46.2)	56 (53.8)	2.30(1.42, 3.74) **	0.63(0.31, 1.29)
	No	61 (27.1)	164 (72.9)	1	1
Previous depression history	Yes	80 (44.9)	98 (55.1)	5.69(3.43, 9.42) ***	5.09(2.77, 9.35) ***
	No	29 (19.2)	122 (80.8)	1	
Social support status	Poor	72 (52.6)	65 (47.4)	5.21(2.43, 11.43)***	5.08(2.15, 11.99) ***
	Moderate	27 (20.2)	108 (80.0)	1.17(0.53, 2.62)	1.24(0.51 3.02)
	Strong	10 (17.5)	47 (82.5)	1	1
Marriage satisfaction status	Dissatisfie d	48 (47.5)	53 (52.5)	2.48(1.52, 4.04) ***	2.37(1.30, 4.33) **
	Satisfied	61 (26.8)	167 (73.2)	1	1

* *p* < 0.05; **p < 0.01; ***p < 0.001; ETB, Ethiopian Birr; COR, crude odds ratio; AOR, adjusted odds ratio

## Discussion

This study assessed the prevalence and associated factors of prenatal depression among pregnant women attending public health facilities in the Babile district, eastern Ethiopia. We found that the prevalence of prenatal depression was 33.1% among pregnant women attending public health facilities in Babile district, eastern Ethiopia.

This finding is consistent with previous studies conducted in Arba Minch, southern Ethiopia (35.4%) [14], west Showa, central Ethiopia (32.3%) [37], Bale, southeast Ethiopia (31.5%) [17], Adama, central Ethiopia (31.2%) [11], and northern Tanzania (33.8%) [38]. However, this finding is higher than those of studies conducted in east Gojam, northwest Ethiopia (17.8%) [39], Addis Ababa, central Ethiopia (24.9%) [25], Hawasa, southern Ethiopia (21.5%) [26], Gondar, northwest Ethiopia (23.0%) [13], Nigeria (24.5%) [35], and Bangladesh (18.0%) [40]. This variation might be due to sociodemographic differences: almost all the study subjects in comparable studies were urban residents [25], while more than half of our study subjects were rural residents. This difference might also be due to differences in the reproductive characteristics of the study subjects. For instance, the rate of contraceptive use was higher in Addis Ababa but lower in our study, which may have resulted in a higher burden of unintended pregnancy associated with prenatal depression. In addition, geographical, cultural, and economic variations may contribute to the higher prevalence of this disease.

On the other hand, the prevalence of depression in this study is lower than that in studies conducted in South Africa (47.0%) [9], Pakistan (81.0%) [41], and Koria (40.5–61.4%) [41]. These variations in prenatal depression could be attributable to differences in the study setting and verification tools used to screen for symptoms of depression and cutoff points for prenatal depression. For instance, the study in Koria used the EPDS with a cutoff point greater than or equal to a score of 9 to diagnose prenatal depression, while our study used a cutoff point greater than or equal to a score of 13.

This study indicated that women with an average monthly income of less than or equal to 2000 ETB were almost four times more likely to develop prenatal depression than women with an income greater than 2000 ETB. These findings imply that lower household income increases the risk of incidental mental disorders. These findings are supported by those of a previous study, which indicated that pregnant women with lower incomes were more likely to develop depression and anxiety [42, 43]. Given that thriving during a healthy pregnancy requires access to adequate nutrition, a safe and reliable place to rest, and other sources of social support.

Women who used contraceptive methods before their current pregnancy were almost 50% less likely to develop prenatal depression during pregnancy than women who did not use contraceptive methods. This finding implies that the use of contraceptives indirectly reduces prenatal depression by reducing the burden of unintended pregnancies. On the other hand, the odds of prenatal depression were greater among women with unintended pregnancies. This could be due to the presence of women who missed opportunities to use modern contraceptive methods for preventing and reducing unintended pregnancy; only a few women may have intended to become pregnant. These women may develop prenatal depression as a result of stress, which is associated with perceiving unintended motherhood with poor social support [44, 45]. It is worrisome that unintended pregnancy could increase the risk of prenatal depression during pregnancy. Reducing the burden of unintended pregnancy through improving the utilization of modern contraceptive methods at the community level is essential for reducing and preventing prenatal depression and its negative consequences.

The odds of prenatal depression were higher among women who had a previous history of depression. This finding is supported by studies conducted in northwestern and central Ethiopia [25, 39]. The risk of depression recurrence is high during pregnancy due to physiological changes. In addition, physical and hormonal changes that occur during pregnancy may precipitate earlier depression, increasing susceptibility to recurrence [23, 25, 46].

Pregnant women who had poor social support were five times more likely to develop prenatal depression than those who had strong social support. This finding is supported by the findings of a study conducted in Hawassa, southern Ethiopia. A poor social life may reduce women’s interaction with others, and it can lead to stress and cause mood changes. This finding is also supported by the findings of previous studies indicating that depression and anxiety are less common among pregnant women who perceive a greater degree of social support [47, 48].

Marriage satisfaction status was significantly associated with prenatal depression. The odds of prenatal depression were about two times higher among women who were dissatisfied with their marriage than among women who were satisfied with their marriage. Marital dissatisfaction leads to marital instability, which may aggravate psychosocial stress and precipitate depression disorder. During pregnancy, women need their husbands’ psychological, physical, and financial support; however, if their marital status is dissatisfied during critical times, they may miss mandatory support and may develop mood instability [49].

As a strength, the study used standard validated instruments for data collection; these instruments can be generalized to pregnant women attending public health facilities in urban and rural settings. However, because of the cross-sectional study design, the study may not have shown a temporal relationship between prenatal depression and the predictors. In addition, because this study was conducted among pregnant women who received antenatal care at health facilities, the findings may not apply to women who did not receive antenatal care.

## Conclusions

This study concluded that one in every three pregnant women attending ANC visits at public health facilities in the Babile district had prenatal depression. Having a lower income, not using contraceptives, unintended pregnancy, a history of previous depression, poor social support, and dissatisfied marriage relations were found to be independent predictors of prenatal depression. Prevention of unintended pregnancies by encouraging women to utilize modern contraceptive methods is essential for mitigating and controlling the risks and burdens of prenatal depression and its negative consequences. In addition, strengthening and building strong social support and healthy marital relationships are paramount for reducing the burden of prenatal depression and its adverse effects.

## Data Availability

Data that support the findings are available and will be provided by the correspondence author on a reasonable request.

## Abbreviations

AOR: Adjusted odds ratio
ANC: Antenatal care
EPDS: Edinburgh Postnatal Depression Scale
LMIC: Low middle-income country
SSA: Sub-Saharan Africa
WHO: World Health Organization
